# A New Strategy Using Rikkunshito to Treat Anorexia and Gastrointestinal Dysfunction

**DOI:** 10.1155/2015/364260

**Published:** 2015-05-03

**Authors:** Yayoi Saegusa, Tomohisa Hattori, Miwa Nahata, Chihiro Yamada, Hiroshi Takeda

**Affiliations:** ^1^Tsumura Research Laboratories, Tsumura & Co., 3586 Yoshiwara, Ami-machi, Inashiki-gun, Ibaraki 300-1192, Japan; ^2^Pathophysiology and Therapeutics, Faculty of Pharmaceutical Sciences, Hokkaido University, Sapporo, Hokkaido 060-0812, Japan; ^3^Department of Gastroenterology and Hepatology, Hokkaido University Graduate School of Medicine, Sapporo, Hokkaido 060-8638, Japan

## Abstract

Because the clinical condition of gastrointestinal dysfunction, including functional dyspepsia, involves tangled combinations of pathologies, there are some cases of insufficient curative efficacy. Thus, traditional herbal medicines (Kampo medicines) uniquely developed in Japan are thought to contribute to medical treatment for upper gastrointestinal symptoms. Rikkunshito is a Kampo medicine often used to treat dyspeptic symptoms. Over the past few years, several studies have investigated the efficacy of rikkunshito for dysmotility, for example, upper abdominal complaints, in animals and humans. Rikkunshito ameliorated the decrease in gastric motility and anorexia in cisplatin-treated rats, stress-loaded mice, and selective serotonin reuptake inhibitor-treated rats by enhancing plasma ghrelin levels via serotonin_2B/2C_ receptor antagonism. In addition, rikkunshito ameliorated the decrease in food intake in aged mice and stress-loaded decreased gastric motility via enhanced ghrelin receptor signaling. Several clinical studies revealed that rikkunshito was effective in ameliorating upper gastrointestinal symptoms, including dyspepsia, epigastric pain, and postprandial fullness. In this review, we discuss these studies and propose additional evidence-based research that may promote the clinical use of Kampo medicines, particularly rikkunshito, for treating anorexia and gastrointestinal dysfunction.

## 1. Introduction

A representative gastrointestinal dysfunction, functional dyspepsia (FD), is associated with symptoms such as gastric pain, anorexia, and postprandial sense of distension. The clinical condition of FD involves numerous factors such as delayed gastric emptying [1], gastric accommodation [2], and psychological factors [3]. The quality of life (QOL) of FD patients is markedly reduced physically, mentally, and socially [4, 5]. In addition, some reports have indicated beneficial therapeutic effects on QOL following improvements in FD symptoms after treatment [6]; thus, the clinical treatment of FD is very important. Although many medications and therapies such as administration of proton-pump inhibitors (PPI), prokinetics, or antidepressants have been attempted, there are some cases of limited curative efficacy. Thus, Kampo medicines have been anticipated to be effective.

**Figure 1:**
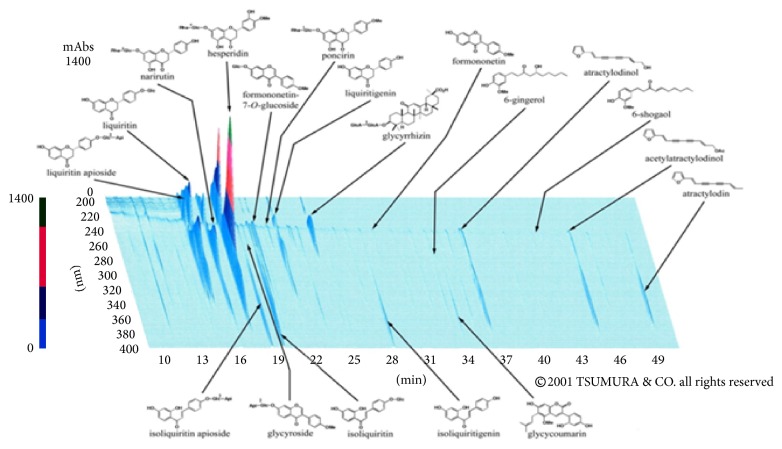
3D-HPLC profiles of rikkunshito components. Data were provided by Tsumura & Co.

Kampo medicines have been uniquely developed in Japan and have been approved by the Ministry of Health, Labour and Welfare of Japan. Clinically, Kampo medicines are used in combination with Western medications or alone. One of these Kampo medicines is rikkunshito, prepared from eight crude drugs: Atractylodis Lanceae Rhizoma, Ginseng Radix, Pinelliae Tuber, Poria, Zizyphi Fructus, Aurantii Nobilis Pericarpium, Glycyrrhizae Radix, and Zingiberis Rhizoma.  Figure 1 shows the UV absorbance characteristics of its herbal ingredients after separation using 3-dimensional high-performance liquid chromatography (3D-HPLC).

In Japan, rikkunshito is commonly used for dyspeptic symptoms [7–9]. It was shown to improve gastrointestinal symptoms in chronic idiopathic dyspepsia patients in a double-blinded, randomized, placebo-controlled trial [10]. In 1998, a large-scale comparative clinical study of 235 patients conducted by Harasawa et al. showed improvement of dyspepsia in dysmotility-like dyspepsia patients after the administration of rikkunshito (the original report was in Japanese and was summarized in English by Hattori [11, 12]). A recent randomized, placebo-controlled trial of rikkunshito for FD patients was conducted by Suzuki et al., and it demonstrated that the administration of rikkunshito reduced dyspepsia and partially improved symptoms of epigastric pain and postprandial fullness in FD patients [13].

Here, we summarize the results of animal studies that investigated the effects of rikkunshito for treating anorexia caused by various factors by focusing on ghrelin, an appetite-promoting hormone. In addition, we discuss the usefulness of treating gastrointestinal disorders such as FD using Kampo medicines, particularly rikkunshito, on the basis of recent clinical studies.

## 2. Gastrointestinal Function-Related Factors: Ghrelin and Serotonin

Ghrelin, a 28-amino-acid peptide, is an orexigenic hormone primarily secreted from X/A-like cells, which are ghrelin-producing cells localized in the stomach mucosa [14]. Ghrelin is found in the blood in two main forms, namely, “acylated ghrelin” and “des-acyl ghrelin,” at a ratio of 1:10. Acylated ghrelin is rapidly metabolized to des-acyl ghrelin by removal of the octanoyl group in blood, which is catalyzed by esterases such as carboxylesterase (CES) in rodents or butyrylcholinesterase (BuChE) in humans [15].

Acylated ghrelin binds to specific receptor, growth hormone secretagogue receptor type 1a (GHS-R1a), localized at the end of the vagus nerve around the stomach [16, 17]. Ghrelin signals are transmitted to the nuclei of the solitary tract and activate neuropeptide Y (NPY)/agouti-related peptide (AgRP) neurons in the hypothalamic arcuate nucleus (ARC) via noradrenergic neurons, resulting in appetite stimulation [16, 17].

Administration of exogenous acylated ghrelin increases food intake in rodents [16]. In addition, acylated ghrelin plays an important role in stomach and duodenal motility [14, 18]. The peak of plasma acylated ghrelin levels is strongly linked with phase III-like contractions in rodents [19]. Exogenous ghrelin administration results in enhanced stomach and duodenal motility [18], leading to accelerated gastric emptying.

Serotonin (5-hydroxytryptamine, 5-HT) plays an important role in various physiological processes, including gastrointestinal function. Central 5-HT plays a role in fear and anxiety manifestations and is involved in appetite regulation. The 5-HT_2_ receptor family is involved in appetite control [20]. 5-HT_2C_ receptors are primarily localized in the brain [21], and 5-HT_2C_ receptor activation induces feeding suppression and anxiety-like behavior in young mice [22–26]. 5-HT_2C_ receptors expressed on proopiomelanocortin (POMC) neurons promote *α*-melanocyte-stimulating hormone production [27], leading to suppression of feeding. Several reports have established that stimulating 5-HT_2C/1B_ receptors by administering *m*-chlorophenylpiperazine (mCPP) induces anorexia in rodents [20, 24, 28–30].

In contrast, 5-HT_2B_ receptors are primarily found in peripheral tissues, including the gastrointestinal tract and stomach fundus [31], and are localized in the brain, as demonstrated recently [32]. Intraperitoneal (IP) administration of BW723C86 (16 mg/kg), a selective 5-HT_2B_ receptor agonist, decreased food intake in rats [33].

IP administration of BW723C86 and mCPP, a 5-HT_2C/1B_ receptor agonist, decreased plasma acylated ghrelin levels in rodents [28]. This suggested that activation of central and/or peripheral 5-HT_2B/2C_ receptors results in decreased ghrelin secretion from X/A-like cells.

## 3. Cisplatin-Induced Anorexia

### 3.1. Cisplatin-Induced Gastrointestinal Disorders

In clinical practice, anticancer drugs such as cisplatin are known to induce gastrointestinal disorders, including acute/delayed nausea, vomiting, anorexia, diarrhea, and weight loss [34]. These markedly affect QOL and may make it difficult to continue chemotherapy. This emetic effect is induced by the activation of 5-HT_3_ receptors [35] in the medulla oblongata owing to the release of large amounts of 5-HT from intestinal enterochromaffin cells [36]. However, the detailed mechanism underlying the loss of appetite because of cisplatin remains unclear.

With regard to anorexia caused by cisplatin, we and others found that in rats treated with cisplatin, there was a decreased 24 h food intake after treatment [28, 37, 38]. Yakabi et al. showed that the decreased food intake caused by IP administration of cisplatin at 4 mg/kg to rats persists up to 48 h after treatment [38].

In both clinical and basic research, recent reports have demonstrated a relationship between anorexia and ghrelin dynamics induced by cisplatin. Some reports have shown that, in humans, plasma ghrelin concentrations decreased during cisplatin-based chemotherapy [39, 40]. In animal studies, we and others showed that cisplatin treatment decreased plasma acylated ghrelin levels in rats [28, 38]. IP administration of 5-HT or cisplatin decreased plasma acylated ghrelin levels in a dose-dependent manner in addition to decreasing the 24 h food intake [28]. Moreover, the reduced plasma acylated ghrelin levels and 24 h food intake following cisplatin treatment could be completely recovered by treatment with 5-HT_2B/2C_ receptor antagonists. In addition, decreased food intake in cisplatin-treated rats could be recovered by exogenous ghrelin treatment. This showed that the reduced plasma acylated ghrelin levels reduced via 5-HT_2B/2C_ receptor activities play a major role in cisplatin-induced anorexia [28]. Interestingly, although plasma acylated ghrelin levels recovered to their baseline levels at 24 h after cisplatin treatment in rats, decreased ghrelin secretion in the hypothalamus persisted even 24 h after treatment, which resulted in a late phase of decreased food intake caused by cisplatin [38]. This suggested that central ghrelin dynamics play an important role in regulating feeding behaviors.

### 3.2. The Effects of Rikkunshito and Its Components on Cisplatin-Induced Anorexia

Rikkunshito administration has been shown to recover decreased food intake and plasma ghrelin levels caused by cisplatin treatment [28, 41]. These effects were also shown to be abolished by administration of [D-Lys^3^]-GHRP-6, a GHS-R antagonist [28, 41]. Thus, the effects of rikkunshito in terms of improving decreased food intake and acylated ghrelin levels in cisplatin-treated rats are likely caused by enhanced ghrelin secretion via 5-HT receptor antagonism, particularly that involving 5-HT_2B/2C_ receptors.

We screened 33 compounds among the many components of rikkunshito for their binding activities with 5-HT receptor subtypes [28]. We found that 3,3′,4′,5,6,7,8-heptamethoxyflavone (HMF), nobiletin, tangeretin (contained in Aurantii Nobilis Pericarpium), and 8-shogaol (contained in Zingiberis Rhizoma) exhibited the strongest inhibitory activity against 5-HT_2B_ receptors; these compounds had inhibition constant (*K*_*i*_) values of 0.21, 0.31, 0.59, and 1.8 *μ*mol/L, respectively. Hesperetin contained in Aurantii Nobilis Pericarpium, the aglycon form of hesperidin, had *K*_*i*_ values of 5.3 *μ*mol/L against 5-HT_2B_ receptors and 20.9 *μ*mol/L against 5-HT_2C_ receptors. Although this inhibitory activity of hesperetin was comparatively weak, the amounts of hesperidin were higher than those of the other compounds tested in our binding assays [42]. Thus, overall, it may exhibit potent 5-HT_2B/2C_ receptor antagonistic activity. Furthermore, hesperetin flavonoids have been reported to enter the brain by passing through the blood-brain barrier [43].

In addition, isoliquiritigenin contained in Glycyrrhizae Radix exhibited the most potent inhibitory activity against 5-HT_2C_ receptor binding (*K*_*i*_ value, 3.5 *μ*mol/L) among all the components tested. In addition, it inhibited 5-HT_2B_ receptor binding inhibitory activity (*K*_*i*_ value, 3.3 *μ*mol/L). Isoliquiritigenin inhibited 5-HT_2C_ receptor activation in a cell functional assay [30]. Furthermore, oral administration of HMF, hesperidin, or isoliquiritigenin in a cisplatin-induced anorexia model resulted in amelioration of the reduced plasma acylated ghrelin levels in a dose-dependent manner [28].

We believe that changes in plasma acylated ghrelin to des-acyl ghrelin (A/D) ratios are also important for regulating feeding behavior. An increase in the A/D ratio after oral administration of rikkunshito in normal control rats and cisplatin-treated rats suggested that rikkunshito inhibits the degradation of acylated ghrelin [44]. We tested 48 rikkunshito components for their inhibitory activities against CES and BuChE and found that 10-gingerol, contained in Zingiberis Rhizoma, had the most potent CES inhibitory activity [44]. We also showed that oral administration of rikkunshito or 10-gingerol increased plasma acylated ghrelin levels and the A/D ratios in acylated ghrelin-treated rats. In addition, administering the CES inhibitor bis(4-nitrophenyl) phosphate resulted in the amelioration of a cisplatin-induced decrease in food intake [44]. These results suggested that the amelioration of cisplatin-induced decreases in food intake and plasma acylated ghrelin levels by rikkunshito is partly attributable to its CES inhibitory effect.

## 4. Stress-Induced Anorexia

### 4.1. Stress and Ghrelin

Stress is a significant social problem [45, 46] known to be associated with anorexia and gastrointestinal function [47, 48]. It has been strongly suggested that stress causes several abnormalities of feeding behavior, such as bulimia and anorexia. In animal studies, food intake reportedly decreases after stress loading, including restraint stress and immobilization stress [49–51] and emotional stress using a communication box [52]. In contrast, increased food intake has been observed after long-term isolation for 3 weeks [53].

Ghrelin levels may also be affected by feeding behaviors of animals under stress. However, there are conflicting data regarding the effects of several stressors on plasma ghrelin levels. Increased plasma ghrelin concentrations were found in a water avoidance stress [54], chronic social defeat stress [55] and repeated restraint stress [56] in rodents, Trier Social Stress Test in humans [57], and cold stress in rodents [58] and humans [59]. In comparison, decreased plasma ghrelin levels have been found to result from immune stress induced by lipopolysaccharide in rodents [60–62], administration of urocortin 1 to rodents [63, 64] and humans [65], and physical exercise at 50% of VO_2max⁡_ in humans [66]. We recently reported that restraint stress causes a significant elevation of plasma des-acyl ghrelin levels only, whereas plasma acylated ghrelin levels remain unaffected [67].

### 4.2. Plasma Ghrelin Levels in Novelty Stressed Mice

One of the stressors that we may experience during daily life is exposure to a new environment. Psychological factors, loneliness, social networks, and environmental changes contribute to decreased food intake, particularly in the elderly [68, 69]. In a novelty stress model, animals are removed from their home cage and placed somewhere they have never been before. This model has been used to estimate anxiety and depression levels [70–72]. We tested the effects of a novel environmental stress on food intake and plasma acylated ghrelin dynamics in young mice [29, 73] and aged mice [30].

We found that novelty stress causes a decrease in food intake, which is associated with decreased plasma ghrelin levels after stress [29]. However, increased plasma ghrelin levels with fasting were not observed in a young mouse novel stress model [73]. Exogenous acylated ghrelin ameliorated the decreased food intake by temporarily increasing plasma acylated ghrelin levels above the physiological concentration [29]. Thus, the transmission of ghrelin signals to the hypothalamic feeding center may be abnormal under novelty stress.

A few studies have investigated a possible relationship between corticotropin-releasing factor (CRF) receptors and plasma ghrelin dynamics. Administration of urocortin 1, a CRF family peptide that binds to both CRF_1_ and CRF_2_ receptors, reduced plasma acylated ghrelin levels in rodents [63, 64]. Yakabi et al. demonstrated that urocortin 1-induced reductions in plasma acylated ghrelin levels and food intake were mediated via CRF_2_ receptors but not CRF_1_ receptors [64]. We reported that novelty stress and CRF administration reduced plasma ghrelin levels and food intake and that a CRF_1_ receptor antagonist but not a CRF_2_ receptor antagonist prevented these decreases [29]. Interestingly, we also found that a selective 5-HT_2C_ or 5-HT_1B_ receptor antagonist and a melanocortin-4 (MC4) receptor antagonist prevented the decreased plasma acylated ghrelin levels in novelty stressed mice [29]. We hypothesized that the acute appetite loss and the decrease in plasma ghrelin levels occurred via CRF_1_ receptors, the effects of which were mediated through 5-HT_2C/1B_ and MC4 receptor systems.

In a novelty stress model, higher levels of central 5-HT and 5-HT receptor expression resulted in the activation of serotonergic signals [72]. 5-HT_2C/1B_ receptor stimulation may downregulate appetite control [25, 74, 75]. We showed that, compared with normal mice, intracerebroventricular administration of mCPP induced a significant decrease in food intake in novelty stressed mice [29]. Administration of 5-HT_2C/1B_ receptor antagonists ameliorated the decrease in food intake and plasma acylated ghrelin levels [29]. Thus, an increase in 5-HT_2C/1B_ receptor activity may occur after novelty stress, resulting in anorexia or reduced plasma ghrelin levels.

In addition, we showed that peripheral administration of SB215505 and SB204741, selective 5-HT_2B_ receptor antagonists, prevented the decrease in food intake in novelty stressed mice [73]. 5-HT_2B_ receptor activation also resulted in decreased food intake [33]. It is therefore possible that 5-HT_2B_ receptors participate in part of the mechanism of action involved in this novelty stress model.

### 4.3. Differential Effects in Aged Mice

It is well known that 5-HT_2C_ receptors are expressed on CRF neurons in the paraventricular nucleus (PVN) and that its activation by 5-HT_2C_ receptor agonists results in adrenocorticotropic hormone (ACTH) secretion [74]. Other studies have shown that CRF mRNA expression and ACTH secretion were enhanced by 5-HT administration to PVN [74, 76] and that mCPP-induced serum corticosterone increases were inhibited by 5-HT_2C_ receptor antagonism [77]. We showed that exposure to a novel environment caused long-term secretion of stress hormones and a continuously decreased food intake in aged mice but not in young mice [30]. In addition, mCPP administration resulted in more severe anorexia in aged control mice than that in young control mice [30]. Thus, the basal level of signal transduction via 5-HT_2C_ receptors may have been enhanced in aged mice.

In our previous report we also found that administering a selective 5-HT_2C_ receptor antagonist, SB242084, to aged mice at a dose that had no effect on food intake in young mice significantly ameliorated both the decrease in food intake and the increase in stress hormone levels after novelty stress [30]. We and others found that novelty stress and social isolation stress enhanced mCPP-responsiveness [29, 71], which may have been linked to upregulated 5-HT_2C/1B_ receptor activity. In addition, we observed increased 5-HT_2C_ receptor gene expression in the hypothalamus at 24 h after novelty stress in aged mice but not in young mice [30]. In summary, we hypothesized that the stimulation or activation of 5-HT_2C_ receptors on CRF neurons in PVN results in activation of the hypothalamic-pituitary-adrenal (HPA) axis in aged mice after novelty stress.

### 4.4. The Effects of Rikkunshito and Its Components on Novelty Stressed Mice

Rikkunshito ameliorated the novelty stress-induced decreases in food intake and plasma ghrelin levels in young mice [29, 73] and in aged mice [30], and coadministering [D-Lys^3^]-GHRP-6 abolished the effects of rikkunshito [29]. Rikkunshito completely ameliorated the decreased food intake in young and aged mice after mCPP injection [30]. Rikkunshito administration attenuated the hyperactivation of the HPA axis and the decreased food intake induced by novelty stress, which was similar to the effects of SB242084 [30]. We and others reported that rikkunshito had an antagonistic effect on 5-HT_2C_ receptors* in vivo* [18, 28]. In addition, the results of* in vitro* radiobinding assays revealed that components in rikkunshito, such as isoliquiritigenin, exhibited 5-HT_2B/2C_ receptor binding inhibitory activity [28]. We also found that glycycoumarin and isoliquiritigenin, which are contained in Glycyrrhizae Radix, ameliorated the reduced food intake in novelty stressed mice [29, 73]. These findings suggest that rikkunshito ameliorates novelty stress-induced anorexia and reduced plasma ghrelin levels via antagonism-like effects on 5-HT_2C_ and 5-HT_2B_ receptors.

### 4.5. The Effects of Rikkunshito on Postprandial Gastric Motility in a Restraint Stress Model

We found that restraint stress decreased the frequency of phase III-like contractions in the fasted state and postprandial gastric contractions in mice [67], leading to delayed gastric emptying. Furthermore, exogenously administered acylated ghrelin and rikkunshito improved the delayed gastric emptying and decreased gastric motility caused by restraint stress, and the rikkunshito effects were completely abolished by a GHS-R antagonist [67]. However, there were no changes in plasma acylated ghrelin levels. Thus, we hypothesized that rikkunshito may have improved the delayed gastric emptying and decreased motility via mechanisms of action other than the enhancing effects on ghrelin release.

Fujitsuka et al. demonstrated that rikkunshito potentiated ghrelin receptor signaling via increased binding between ghrelin and ghrelin receptors [78]. Thus, exogenous ghrelin supplementation or ghrelin signal enhancement by rikkunshito may be effective for improving symptoms in FD patients.

## 5. Aging-Induced Anorexia

### 5.1. Anorexia-Associated Malnutrition in the Elderly

In the elderly, malnutrition can cause various problems such as problems related to daily life activities, reduced immune function, and loss of muscle strength [79–81]. Therefore, dealing with malnutrition is quite important. Anorexia is the main cause of malnutrition in the elderly [82]. Food intake has been shown to decrease gradually with age [82]. Various factors are responsible for anorexia in the elderly, including social isolation, diseases such as depression and physical disorders, reduced gustatory and olfactory senses, and medicines [83].

Appetite is controlled by central and peripheral orexigenic/anorexigenic factors [84]. As a central control mechanism, NPY and AgRP levels are altered with aging [85–88] and NPY signaling is dysfunctional in old rats [89]. However, few reports regarding the changes in neurotransmitters of the central nervous system that accompany aging in humans are available.

The elderly have lower levels of plasma ghrelin than the young people, and ghrelin secretion from the stomach decreases with aging [90, 91]. However, some reports have shown that there were no differences in the ghrelin levels between young and aged humans [92] and mice [93], which reflects controversy with regard to age-associated changes in ghrelin dynamics.

### 5.2. Ghrelin Resistance and Hyperleptinemia in Aged Mice

In animal models, 24 h food intake and 2-week body weight gain decreased in aged mice compared with young mice [94]. Our results showed that the plasma ghrelin levels in aged mice did not increase while fasting and that the levels were higher while feeding than those in young mice [94]. These results prompted us to conclude that the regulation of ghrelin secretion may be disturbed in aged mice. Moreover, exogenous ghrelin administration markedly enhanced food intake in young mice but not in aged mice [94]. Thus, ghrelin signaling may be impaired in aged mice.

Leptin, an adipocyte-derived hormone, suppresses food intake and decreases body adiposity [95]. We found that plasma leptin levels in aged mice were very high and this increased plasma leptin level was maintained regardless of ingestion [94]. In ARC, leptin receptors are expressed on NPY neurons and POMC neurons [96, 97], and GHS-R is expressed on NPY neurons [98]. Ghrelin and leptin may have opposing actions on NPY neurons; thus, abnormally high concentrations of leptin are considered to reduce the effects of ghrelin [99]. Another report showed that hyperleptinemia prevented an increase in ghrelin levels [100].

It was also suggested that leptin suppressed ghrelin signaling by NPY neurons via the activation of the phosphoinositide 3-kinase- (PI3K-) phosphodiesterase 3 (PDE3) pathway, which may have abolished the adenylate cyclase-cAMP-protein kinase A system implicated in the effects of ghrelin [101]. We found that the administration of a PI3K inhibitor and a PDE3 inhibitor ameliorated the anorexia in aged mice [94]. Thus, we propose that the hyperleptinemia accompanying aging may induce resistance to ghrelin reactivity in aged mice by downregulating cAMP levels [94].

### 5.3. The Effects of Rikkunshito and Its Components on Anorexia in Aged Mice

We showed that the administration of rikkunshito could ameliorate some effects of aging-associated anorexia [94]. Exogenous ghrelin ameliorated decreased food intake in a cisplatin-induced anorexia model [28] and a novelty stress-induced anorexia model [29, 73] but not in our aging-anorexia model [94]. After administering rikkunshito, increased plasma ghrelin levels were not observed in aged mice; thus, increased ghrelin secretion was not the main mechanism underlying the amelioration caused by rikkunshito.

We tested 33 components of rikkunshito and found that HMF, nobiletin, isoliquiritigenin, and glycycoumarin exhibited inhibitory effects on PDE3 activity. It was previously reported that nobiletin flavonoids could enter the brain by passing through the blood-brain barrier [102]. Thus, these results suggested that rikkunshito ameliorates aging-induced anorexia via enhanced ghrelin receptor signaling by PDE3 inhibition.

## 6. Clinical Applications of Rikkunshito

FD is likely to occur through the combined effects of different pathologies. As described in this paper, the results of animal studies suggest that rikkunshito enhances appetite and gastric motility [18, 67] by increasing endogenous ghrelin levels [18, 28, 29, 73, 103] or ghrelin signals [78, 94] and thereby ameliorates upper gastrointestinal dysfunctions, including FD. Studies of healthy human volunteers [103, 104] and FD patients [105] have shown that endogenous acylated ghrelin levels increase after rikkunshito administration.

In a clinical study conducted by Arai et al. using a parallel, randomized, controlled trial of gastroprokinetic agents for 27 patients, it was shown that rikkunshito was effective in ameliorating upper gastrointestinal symptoms, as evaluated by their scores on the Gastrointestinal Symptom Rating Scale questionnaire [105]. Tominaga et al. conducted a randomized, placebo-controlled, double-blind clinical trial of rikkunshito for 242 patients with nonerosive reflux disease refractory to PPI [106]. Treatment for 4 weeks with rikkunshito significantly improved their mental component summary (MCS) scores in the Short-Form Health Survey-8 (SF-8). After 8 weeks of treatment with rikkunshito, MCS scores in SF-8 improved in patients with low body mass index values (<22), and acid-related dysmotility symptoms assessed by the Frequency Scale for the Symptoms of Gastroesophageal Reflux Disease also improved in females and the elderly. Another clinical trial was conducted by Suzuki et al.; it was a multicenter, randomized, double-blind, placebo-controlled, parallel-group trial on the effect of rikkunshito on 247 patients [13]. Administration of rikkunshito for 8 weeks reduced dyspepsia, epigastric pain was significantly improved, and postprandial fullness tended to improve.

Anorexia is a cause of concern for cancer patients since a persistent loss of appetite develops into cancer cachexia. A clinical trial of ghrelin receptor agonists has revealed that there is a remarkable effect on weight gain in patients with non-small-cell lung cancer [107]. It has been confirmed that rikkunshito also improves QOL in advanced esophageal cancer patients [108] and prolongs survival in stage III/IV pancreatic cancer patients and tumor-bearing rats [78]. Unlike other ghrelin receptor agonists, rikkunshito displays multiple actions related to ghrelin signal activation, that is, stimulation of ghrelin secretion and sustained activity of GHS-R, and prevention of the degradation of endogenous acylated ghrelin. Therefore, it is expected that rikkunshito may be effective to the ghrelin resistance seen in cancer anorexia-cachexia [78]. Further, rikkunshito is potentially effective in improving gastrointestinal symptoms in patients after gastrectomy [109, 110]. However, since there are few reports in patients with cancer cachexia or with upper gastrointestinal surgery, further large-scale clinical trials are required.

Evidence of the relevance of using rikkunshito to treat anorexia and gastrointestinal dysfunction continues to accumulate, as summarized here. In addition, the use of Kampo medicines as therapeutic agents for FD has recently been proposed in Japan (guidelines for functional gastrointestinal diseases: 2014). With continuing evidence-based high-quality research, the mechanisms of action of Kampo medicines, particularly those of rikkunshito, may be elucidated to a greater extent, and the use of Kampo medicines may expand as a front line treatment for anorexia and gastrointestinal dysfunction.
